# 1-[3-({[Bis(2-methyl­prop­yl)carbamo­thio­yl]amino}­carbon­yl)benzoyl]-3,3-bis­(2-methyl­prop­yl)thio­urea

**DOI:** 10.1107/S1600536813021053

**Published:** 2013-08-03

**Authors:** N. Selvakumaran, R. Karvembu, Seik Weng Ng, Edward R. T. Tiekink

**Affiliations:** a Department of Chemistry, National Institute of Technology, Tiruchirappalli 620 015, India; b Department of Chemistry, University of Malaya, 50603 Kuala Lumpur, Malaysia; c Chemistry Department, Faculty of Science, King Abdulaziz University, PO Box 80203 Jeddah, Saudi Arabia

## Abstract

The title compound, C_26_H_42_N_4_O_2_S_2_, adopts a shallow U-shape as both pendant arms of the 1,3-substituted benzene ring are orientated in the same direction. The thione S atoms lie to the same side of the benzene ring and the carbonyl O atoms to the other. The most prominent feature of the crystal packing is the formation of inversion dimers mediated by N—H⋯S hydrogen bonds. One of the 2-methyl­propyl groups is statistically disordered over two positions.

## Related literature

For the use of the title compound in the synthesis of metal macrocycles, see: Nguyen *et al.* (2011 ▶). For the structure of the methanol solvate, see: Rodenstein *et al.* (2007 ▶).
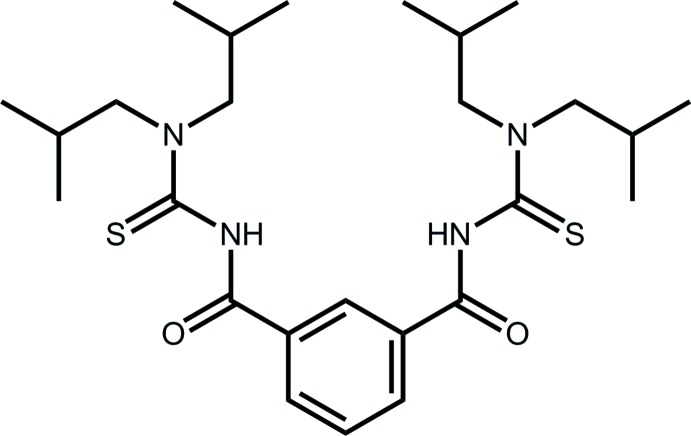


## Experimental

### 

#### Crystal data

C_26_H_42_N_4_O_2_S_2_
*M* *_r_* = 506.76
Monoclinic, 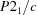

*a* = 12.6926 (2) Å
*b* = 11.8015 (2) Å
*c* = 19.9701 (4) Å
β = 103.883 (2)°
*V* = 2903.97 (9) Å^3^
*Z* = 4
Cu *K*α radiation
μ = 1.87 mm^−1^
*T* = 100 K
0.30 × 0.25 × 0.20 mm

#### Data collection

Agilent SuperNova Dual diffractometer with an Atlas detector
Absorption correction: multi-scan (*CrysAlis PRO*; Agilent, 2013 ▶) *T*_min_ = 0.217, *T*_max_ = 1.000
11629 measured reflections
5743 independent reflections
5234 reflections with *I* > 2σ(*I*)
*R*_int_ = 0.028

#### Refinement

*R*[*F* ^2^ > 2σ(*F* ^2^)] = 0.050
*wR*(*F* ^2^) = 0.142
*S* = 1.01
5743 reflections
316 parameters
30 restraints
H-atom parameters constrained
Δρ_max_ = 0.89 e Å^−3^
Δρ_min_ = −0.82 e Å^−3^

### 

Data collection: *CrysAlis PRO* (Agilent, 2013 ▶); cell refinement: *CrysAlis PRO*; data reduction: *CrysAlis PRO*; program(s) used to solve structure: *SHELXS97* (Sheldrick, 2008 ▶); program(s) used to refine structure: *SHELXL97* (Sheldrick, 2008 ▶); molecular graphics: *ORTEP-3 for Windows* (Farrugia, 2012 ▶) and *DIAMOND* (Brandenburg, 2006 ▶); software used to prepare material for publication: *publCIF* (Westrip, 2010 ▶).

## Supplementary Material

Crystal structure: contains datablock(s) general, I. DOI: 10.1107/S1600536813021053/hg5336sup1.cif

Structure factors: contains datablock(s) I. DOI: 10.1107/S1600536813021053/hg5336Isup2.hkl

Supplementary material file. DOI: 10.1107/S1600536813021053/hg5336Isup3.cml

Additional supplementary materials:  crystallographic information; 3D view; checkCIF report

## Figures and Tables

**Table 1:** Hydrogen-bond geometry (Å, °)

*D*—H⋯*A*	*D*—H	H⋯*A*	*D*⋯*A*	*D*—H⋯*A*
N2—H2⋯S2^i^	0.88	2.62	3.4745 (17)	163
N3—H3⋯S2^i^	0.88	2.54	3.3870 (18)	162

Symmetry code: (i) 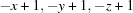.

**Fig. 1.**
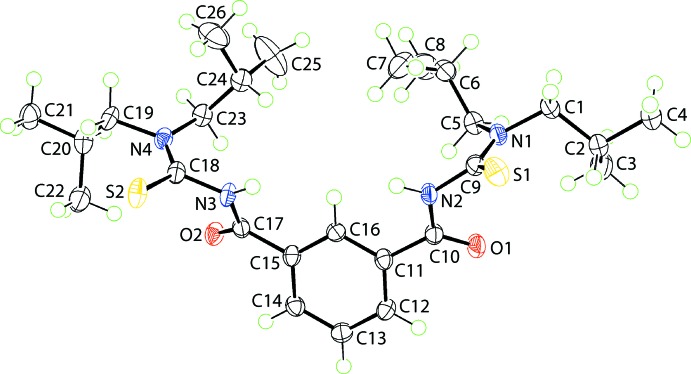
Molecular structure of (I) showing atom-labelling scheme and displacement ellipsoids at the 70% probability level.

**Fig. 2.**
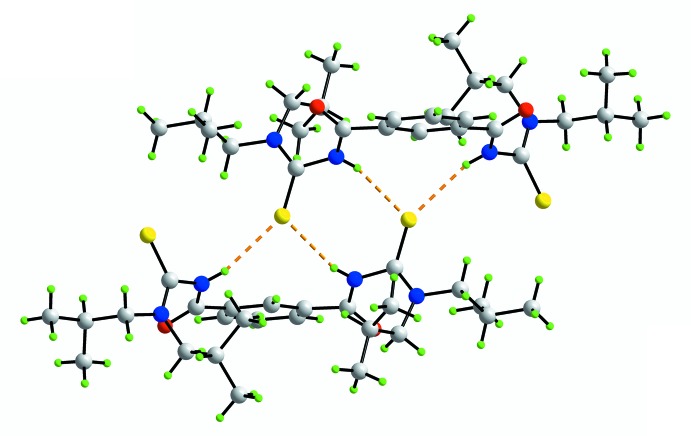
A view of the supramolecular dimer sustained by N—H···S hydrogen bonding, shown as orange dashed lines.

## supplementary crystallographic information

### 1. Comment

The title compound, (I), for which the structure of a methanol solvate is known (Rodenstein *et al.*, 2007), plays a vital role in forming a metallamacrocyclic square-planar *d*^8^ metal complexes *via* self-assembly (Nguyen *et al.*, 2011).

In (I), Fig. 1, there is an almost orthogonal relationship between adjacent thione and carbonyl groups as seen in the C10—N2—C9—S1 and C17—N3—C18—S2 torsion angles of -113.25 (18) and 112.56 (18)°, respectively. Globally, the molecule adopts a flattened U-shape. The thione-S atoms lie to one side of the plane through the central benzene and the carbonyl-O atoms to the other so that the molecule has approximate mirror symmetry. The structure reported here for (I) is quite distinct that that observed in the methanol solvate of (I) (Rodenstein *et al.*, 2007). In the latter, the thione-S atoms lie to either side of the benzene ring and, to a first approximation, the carbonyl-O atoms are co-planar with the ring.

Being direct towards the centre of the U-shaped molecule, the nitrogen-bound hydrogen atoms are well placed to form two N—H···S hydrogen bonds with an inverted U-shaped molecule with the central pair forming an eight-membered {···HNCS}_2_ synthon, Fig. 2 and Table 1. A detailed analysis of the crystal packing is precluded owing to the presence of disorder in the molecule.

### 2. Experimental

Isophthaloyl dichloride (2.0302 g, 10 mmol) dissolved in acetone (80 ml) was placed in a dropping funnel and added drop wise with stirring to potassium thiocyanate (1.9436 g, 20 mmol) dissolved in acetone (80 ml), under an N_2_ atmosphere, in a three-necked round bottom flask. The mixture was heated to reflux for 30 min. and then allowed to cool. A solution of diisobutylamine (2.2850 g, 20 mmol) in acetone (80 ml) was added drop wise from a dropping funnel to the reaction mixture and the resulting mixture was stirred for 2 h. Hydrochloric acid (0.1 N, 300 ml) was added and the resulting white solid was filtered off, washed with water and dried in *vacuo*. Single crystals were grown at room temperature from its acetonitrile solution. FT—IR (KBr): ν(N—H) 3275, ν(C═O) 1694, ν(C═C) 1604, ν(C═S) 1262 cm^-1^.

### 3. Refinement

Carbon-bound H-atoms were placed in calculated positions [N—H = 0.88 Å; C—H = 0.95–0.99 Å, *U*_iso_(H) = 1.2–1.5*U*_eq_(N, C)] and were included in the refinement in the riding model approximation. The C24-containing groups was disordered over two positions of equal weight, from refinement. Pairs of 1,2-related distances were restrained to 1.50±0.01 Å and the 1,3-related ones to 2.35±0.01 Å. The anisotropic displacement parameters (adp) of the primed atoms were set to those of the unprimed ones, and the adp of the three components of the affected atoms were tightly restrained with the ISOR command in *SHELXL97*.

### Crystal data

**Table:**

C_26_H_42_N_4_O_2_S_2_	*F*(000) = 1096
*M**_r_* = 506.76	*D*_x_ = 1.159 Mg m^−^^3^
Monoclinic, *P*2_1_/*c*	Cu *K*α radiation, λ = 1.54184 Å
Hall symbol: -P 2ybc	Cell parameters from 7250 reflections
*a* = 12.6926 (2) Å	θ = 3.6–74.2°
*b* = 11.8015 (2) Å	µ = 1.87 mm^−^^1^
*c* = 19.9701 (4) Å	*T* = 100 K
β = 103.883 (2)°	Prism, colourless
*V* = 2903.97 (9) Å^3^	0.30 × 0.25 × 0.20 mm
*Z* = 4	

### Data collection

**Table:**

Agilent SuperNova Dual diffractometer with an Atlas detector	5743 independent reflections
Radiation source: SuperNova (Cu) X-ray Source	5234 reflections with *I* > 2σ(*I*)
Mirror monochromator	*R*_int_ = 0.028
Detector resolution: 10.4041 pixels mm^-1^	θ_max_ = 74.4°, θ_min_ = 3.6°
ω scan	*h* = −15→13
Absorption correction: multi-scan (*CrysAlis PRO*; Agilent, 2013)	*k* = −12→14
*T*_min_ = 0.217, *T*_max_ = 1.000	*l* = −24→24
11629 measured reflections	

### Refinement

**Table:**

Refinement on *F*^2^	Primary atom site location: structure-invariant direct methods
Least-squares matrix: full	Secondary atom site location: difference Fourier map
*R*[*F*^2^ > 2σ(*F*^2^)] = 0.050	Hydrogen site location: inferred from neighbouring sites
*wR*(*F*^2^) = 0.142	H-atom parameters constrained
*S* = 1.01	*w* = 1/[σ^2^(*F*_o_^2^) + (0.0843*P*)^2^ + 2.364*P*] where *P* = (*F*_o_^2^ + 2*F*_c_^2^)/3
5743 reflections	(Δ/σ)_max_ = 0.001
316 parameters	Δρ_max_ = 0.89 e Å^−^^3^
30 restraints	Δρ_min_ = −0.82 e Å^−^^3^

### Special details

**Table:**

Geometry. All e.s.d.'s (except the e.s.d. in the dihedral angle between two l.s. planes) are estimated using the full covariance matrix. The cell e.s.d.'s are taken into account individually in the estimation of e.s.d.'s in distances, angles and torsion angles; correlations between e.s.d.'s in cell parameters are only used when they are defined by crystal symmetry. An approximate (isotropic) treatment of cell e.s.d.'s is used for estimating e.s.d.'s involving l.s. planes.
Refinement. Refinement of *F*^2^ against ALL reflections. The weighted *R*-factor *wR* and goodness of fit *S* are based on *F*^2^, conventional *R*-factors *R* are based on *F*, with *F* set to zero for negative *F*^2^. The threshold expression of *F*^2^ > σ(*F*^2^) is used only for calculating *R*-factors(gt) *etc*. and is not relevant to the choice of reflections for refinement. *R*-factors based on *F*^2^ are statistically about twice as large as those based on *F*, and *R*- factors based on ALL data will be even larger.

### Fractional atomic coordinates and isotropic or equivalent isotropic displacement parameters (Å^2^)

**Table:**

	*x*	*y*	*z*	*U*_iso_*/*U*_eq_	Occ. (<1)
S1	0.11609 (4)	0.55881 (4)	0.67655 (3)	0.02477 (15)	
S2	0.62752 (4)	0.46118 (4)	0.44878 (3)	0.02293 (15)	
O1	0.17340 (12)	0.22802 (13)	0.68690 (8)	0.0241 (3)	
O2	0.61704 (11)	0.15808 (12)	0.54351 (7)	0.0206 (3)	
N1	0.24077 (13)	0.43431 (13)	0.77683 (8)	0.0169 (3)	
N2	0.25286 (13)	0.39332 (14)	0.66500 (8)	0.0170 (3)	
H2	0.2964	0.4261	0.6426	0.020*	
N3	0.56570 (13)	0.34366 (14)	0.54531 (9)	0.0176 (3)	
H3	0.5191	0.3901	0.5572	0.021*	
N4	0.75240 (13)	0.37320 (13)	0.56575 (9)	0.0178 (3)	
C1	0.18861 (16)	0.48806 (17)	0.82699 (10)	0.0203 (4)	
H1A	0.1745	0.5688	0.8145	0.024*	
H1B	0.2388	0.4851	0.8733	0.024*	
C2	0.08139 (17)	0.43044 (18)	0.82988 (11)	0.0219 (4)	
H2A	0.0328	0.4316	0.7824	0.026*	
C3	0.0975 (2)	0.3077 (2)	0.85294 (13)	0.0306 (5)	
H3A	0.1339	0.2665	0.8223	0.046*	
H3B	0.0268	0.2729	0.8511	0.046*	
H3C	0.1421	0.3046	0.9003	0.046*	
C4	0.02682 (19)	0.4997 (2)	0.87688 (13)	0.0323 (5)	
H4A	−0.0413	0.4630	0.8796	0.048*	
H4B	0.0116	0.5763	0.8580	0.048*	
H4C	0.0752	0.5042	0.9231	0.048*	
C5	0.33477 (16)	0.36086 (17)	0.80564 (10)	0.0196 (4)	
H5A	0.3458	0.3086	0.7691	0.023*	
H5B	0.3183	0.3143	0.8431	0.023*	
C6	0.44031 (17)	0.42639 (19)	0.83420 (11)	0.0239 (4)	
H6	0.4305	0.4746	0.8734	0.029*	
C7	0.47084 (19)	0.5028 (2)	0.78033 (13)	0.0334 (5)	
H7A	0.5390	0.5420	0.8008	0.050*	
H7B	0.4132	0.5587	0.7643	0.050*	
H7C	0.4800	0.4568	0.7412	0.050*	
C8	0.52897 (19)	0.3391 (2)	0.86208 (13)	0.0347 (5)	
H8A	0.5980	0.3782	0.8804	0.052*	
H8B	0.5363	0.2881	0.8248	0.052*	
H8C	0.5094	0.2952	0.8990	0.052*	
C9	0.20600 (16)	0.45859 (16)	0.71007 (10)	0.0177 (4)	
C10	0.23093 (15)	0.27912 (16)	0.65600 (10)	0.0173 (4)	
C11	0.28645 (15)	0.22035 (16)	0.60795 (10)	0.0164 (4)	
C12	0.23975 (16)	0.12179 (16)	0.57527 (10)	0.0183 (4)	
H12	0.1725	0.0956	0.5824	0.022*	
C13	0.29103 (16)	0.06192 (16)	0.53250 (10)	0.0196 (4)	
H13	0.2580	−0.0040	0.5093	0.024*	
C14	0.39096 (16)	0.09851 (16)	0.52349 (10)	0.0179 (4)	
H14	0.4266	0.0568	0.4947	0.021*	
C15	0.43890 (15)	0.19632 (16)	0.55670 (10)	0.0162 (4)	
C16	0.38622 (15)	0.25765 (16)	0.59863 (9)	0.0158 (4)	
H16	0.4182	0.3248	0.6208	0.019*	
C17	0.54908 (15)	0.22891 (16)	0.54792 (10)	0.0164 (4)	
C18	0.65585 (16)	0.38956 (16)	0.52398 (10)	0.0173 (4)	
C19	0.85265 (15)	0.40405 (17)	0.54486 (11)	0.0200 (4)	
H19A	0.8997	0.4496	0.5819	0.024*	
H19B	0.8337	0.4515	0.5028	0.024*	
C20	0.91545 (16)	0.29955 (17)	0.53062 (11)	0.0217 (4)	
H20	0.9352	0.2529	0.5736	0.026*	
C21	1.02006 (16)	0.33990 (19)	0.51283 (11)	0.0252 (4)	
H21A	1.0612	0.3873	0.5504	0.038*	
H21B	1.0020	0.3841	0.4700	0.038*	
H21C	1.0639	0.2741	0.5067	0.038*	
C22	0.84824 (19)	0.22679 (19)	0.47291 (13)	0.0292 (5)	
H22A	0.7817	0.2023	0.4854	0.044*	
H22B	0.8904	0.1601	0.4660	0.044*	
H22C	0.8294	0.2711	0.4302	0.044*	
C23	0.76747 (16)	0.33369 (17)	0.63803 (10)	0.0208 (4)	
H23A	0.7282	0.2611	0.6375	0.025*	0.50
H23B	0.8456	0.3181	0.6570	0.025*	0.50
H23C	0.8314	0.2830	0.6501	0.025*	0.50
H23D	0.7030	0.2898	0.6423	0.025*	0.50
C24	0.7304 (4)	0.4126 (4)	0.6856 (2)	0.0249 (7)	0.50
H24	0.6498	0.4219	0.6718	0.030*	0.50
C25	0.7662 (18)	0.3648 (9)	0.7587 (4)	0.0535 (12)	0.50
H25A	0.7500	0.2835	0.7580	0.080*	0.50
H25B	0.7272	0.4038	0.7888	0.080*	0.50
H25C	0.8445	0.3764	0.7763	0.080*	0.50
C26	0.7866 (5)	0.5259 (4)	0.6858 (3)	0.0385 (8)	0.50
H26A	0.7744	0.5723	0.7239	0.058*	0.50
H26B	0.7571	0.5649	0.6420	0.058*	0.50
H26C	0.8647	0.5138	0.6916	0.058*	0.50
C24'	0.7841 (4)	0.4354 (4)	0.6890 (2)	0.0249 (7)	0.50
H24'	0.8598	0.4653	0.6964	0.030*	0.50
C25'	0.7669 (18)	0.3828 (10)	0.7565 (4)	0.0535 (12)	0.50
H25D	0.7828	0.4395	0.7934	0.080*	0.50
H25E	0.8156	0.3178	0.7695	0.080*	0.50
H25F	0.6915	0.3576	0.7494	0.080*	0.50
C26'	0.7058 (5)	0.5276 (4)	0.6693 (3)	0.0385 (8)	0.50
H26D	0.7060	0.5536	0.6227	0.058*	0.50
H26E	0.7257	0.5905	0.7019	0.058*	0.50
H26F	0.6331	0.5003	0.6700	0.058*	0.50

### Atomic displacement parameters (Å^2^)

**Table:**

	*U*^11^	*U*^22^	*U*^33^	*U*^12^	*U*^13^	*U*^23^
S1	0.0289 (3)	0.0230 (3)	0.0233 (3)	0.00895 (19)	0.0080 (2)	0.00262 (19)
S2	0.0208 (3)	0.0234 (3)	0.0279 (3)	0.00407 (18)	0.0124 (2)	0.00993 (19)
O1	0.0282 (8)	0.0239 (7)	0.0252 (7)	−0.0074 (6)	0.0163 (6)	−0.0031 (6)
O2	0.0200 (7)	0.0164 (7)	0.0286 (8)	0.0007 (5)	0.0119 (6)	−0.0014 (5)
N1	0.0179 (8)	0.0179 (8)	0.0167 (8)	0.0007 (6)	0.0078 (6)	−0.0014 (6)
N2	0.0189 (8)	0.0175 (8)	0.0175 (8)	−0.0016 (6)	0.0101 (6)	−0.0002 (6)
N3	0.0165 (8)	0.0143 (8)	0.0255 (9)	0.0001 (6)	0.0121 (7)	0.0015 (6)
N4	0.0187 (8)	0.0141 (7)	0.0228 (8)	−0.0011 (6)	0.0092 (6)	0.0005 (6)
C1	0.0227 (10)	0.0206 (10)	0.0200 (9)	−0.0001 (8)	0.0098 (8)	−0.0042 (8)
C2	0.0219 (10)	0.0248 (10)	0.0217 (10)	−0.0019 (8)	0.0106 (8)	−0.0042 (8)
C3	0.0328 (12)	0.0269 (11)	0.0376 (13)	−0.0042 (9)	0.0190 (10)	0.0012 (9)
C4	0.0283 (11)	0.0371 (13)	0.0369 (13)	−0.0012 (10)	0.0186 (10)	−0.0105 (10)
C5	0.0212 (10)	0.0207 (10)	0.0180 (9)	0.0021 (8)	0.0071 (8)	0.0010 (7)
C6	0.0206 (10)	0.0299 (11)	0.0215 (10)	0.0002 (8)	0.0052 (8)	−0.0059 (8)
C7	0.0249 (11)	0.0387 (13)	0.0370 (13)	−0.0099 (10)	0.0085 (10)	−0.0008 (10)
C8	0.0256 (11)	0.0425 (14)	0.0326 (12)	0.0074 (10)	0.0002 (9)	−0.0060 (10)
C9	0.0188 (9)	0.0161 (9)	0.0202 (9)	−0.0026 (7)	0.0086 (8)	−0.0020 (7)
C10	0.0170 (9)	0.0193 (9)	0.0168 (9)	−0.0005 (7)	0.0063 (7)	0.0005 (7)
C11	0.0189 (9)	0.0156 (9)	0.0163 (9)	−0.0002 (7)	0.0070 (7)	0.0023 (7)
C12	0.0167 (9)	0.0177 (9)	0.0216 (9)	−0.0022 (7)	0.0065 (7)	0.0009 (7)
C13	0.0215 (10)	0.0157 (9)	0.0221 (10)	−0.0032 (7)	0.0058 (8)	−0.0023 (7)
C14	0.0202 (9)	0.0159 (9)	0.0187 (9)	0.0010 (7)	0.0071 (7)	−0.0012 (7)
C15	0.0175 (9)	0.0151 (9)	0.0171 (9)	−0.0002 (7)	0.0066 (7)	0.0029 (7)
C16	0.0182 (9)	0.0134 (8)	0.0164 (9)	−0.0017 (7)	0.0052 (7)	−0.0002 (7)
C17	0.0190 (9)	0.0157 (9)	0.0162 (9)	−0.0010 (7)	0.0078 (7)	−0.0005 (7)
C18	0.0192 (9)	0.0122 (8)	0.0235 (10)	0.0000 (7)	0.0110 (7)	−0.0003 (7)
C19	0.0160 (9)	0.0181 (9)	0.0285 (10)	−0.0021 (7)	0.0104 (8)	0.0025 (8)
C20	0.0220 (10)	0.0202 (10)	0.0261 (10)	0.0045 (8)	0.0118 (8)	0.0060 (8)
C21	0.0174 (10)	0.0325 (11)	0.0266 (11)	0.0029 (8)	0.0073 (8)	0.0022 (9)
C22	0.0303 (11)	0.0215 (10)	0.0416 (13)	−0.0044 (9)	0.0202 (10)	−0.0055 (9)
C23	0.0198 (9)	0.0217 (10)	0.0217 (10)	−0.0007 (8)	0.0067 (8)	0.0020 (8)
C24	0.026 (2)	0.0248 (17)	0.0264 (14)	−0.0041 (16)	0.0106 (18)	−0.0030 (13)
C25	0.072 (2)	0.066 (3)	0.0243 (14)	0.030 (3)	0.0160 (14)	0.0035 (15)
C26	0.050 (2)	0.0312 (17)	0.0356 (19)	0.0013 (19)	0.0119 (18)	−0.0110 (15)
C24'	0.026 (2)	0.0248 (17)	0.0264 (14)	−0.0041 (16)	0.0106 (18)	−0.0030 (13)
C25'	0.072 (2)	0.066 (3)	0.0243 (14)	0.030 (3)	0.0160 (14)	0.0035 (15)
C26'	0.050 (2)	0.0312 (17)	0.0356 (19)	0.0013 (19)	0.0119 (18)	−0.0110 (15)

### Geometric parameters (Å, º)

**Table:**

S1—C9	1.668 (2)	C12—H12	0.9500
S2—C18	1.685 (2)	C13—C14	1.392 (3)
O1—C10	1.222 (2)	C13—H13	0.9500
O2—C17	1.219 (2)	C14—C15	1.396 (3)
N1—C9	1.331 (3)	C14—H14	0.9500
N1—C1	1.470 (2)	C15—C16	1.393 (3)
N1—C5	1.475 (2)	C15—C17	1.501 (3)
N2—C10	1.379 (3)	C16—H16	0.9500
N2—C9	1.419 (2)	C19—C20	1.532 (3)
N2—H2	0.8800	C19—H19A	0.9900
N3—C17	1.374 (2)	C19—H19B	0.9900
N3—C18	1.421 (2)	C20—C22	1.524 (3)
N3—H3	0.8800	C20—C21	1.530 (3)
N4—C18	1.321 (3)	C20—H20	1.0000
N4—C19	1.477 (2)	C21—H21A	0.9800
N4—C23	1.485 (3)	C21—H21B	0.9800
C1—C2	1.535 (3)	C21—H21C	0.9800
C1—H1A	0.9900	C22—H22A	0.9800
C1—H1B	0.9900	C22—H22B	0.9800
C2—C3	1.519 (3)	C22—H22C	0.9800
C3—H3A	0.9800	C23—C24	1.485 (4)
C3—H3B	0.9800	C23—C24'	1.556 (4)
C3—H3C	0.9800	C23—H23A	0.9900
C2—C4	1.530 (3)	C23—H23B	0.9900
C2—H2A	1.0000	C23—H23C	0.9900
C4—H4A	0.9800	C23—H23D	0.9900
C4—H4B	0.9800	C24—C26	1.515 (6)
C4—H4C	0.9800	C24—C25	1.529 (8)
C5—C6	1.533 (3)	C24—H24	1.0000
C5—H5A	0.9900	C25—H25A	0.9800
C5—H5B	0.9900	C25—H25B	0.9800
C6—C7	1.523 (3)	C25—H25C	0.9800
C6—C8	1.528 (3)	C26—H26A	0.9800
C6—H6	1.0000	C26—H26B	0.9800
C7—H7A	0.9800	C26—H26C	0.9800
C7—H7B	0.9800	C24'—C26'	1.463 (6)
C7—H7C	0.9800	C24'—C25'	1.547 (8)
C8—H8A	0.9800	C24'—H24'	1.0000
C8—H8B	0.9800	C25'—H25D	0.9800
C8—H8C	0.9800	C25'—H25E	0.9800
C10—C11	1.491 (3)	C25'—H25F	0.9800
C11—C16	1.395 (3)	C26'—H26D	0.9800
C11—C12	1.395 (3)	C26'—H26E	0.9800
C12—C13	1.385 (3)	C26'—H26F	0.9800
			
C9—N1—C1	119.61 (16)	N3—C17—C15	114.39 (16)
C9—N1—C5	124.04 (16)	N4—C18—N3	116.55 (17)
C1—N1—C5	116.23 (15)	N4—C18—S2	127.12 (15)
C10—N2—C9	120.73 (16)	N3—C18—S2	116.32 (14)
C10—N2—H2	119.6	N4—C19—C20	112.09 (16)
C9—N2—H2	119.6	N4—C19—H19A	109.2
C17—N3—C18	121.97 (16)	C20—C19—H19A	109.2
C17—N3—H3	119.0	N4—C19—H19B	109.2
C18—N3—H3	119.0	C20—C19—H19B	109.2
C18—N4—C19	121.16 (17)	H19A—C19—H19B	107.9
C18—N4—C23	122.92 (16)	C22—C20—C21	111.05 (17)
C19—N4—C23	115.67 (16)	C22—C20—C19	111.87 (17)
N1—C1—C2	112.50 (16)	C21—C20—C19	108.17 (17)
N1—C1—H1A	109.1	C22—C20—H20	108.6
C2—C1—H1A	109.1	C21—C20—H20	108.6
N1—C1—H1B	109.1	C19—C20—H20	108.6
C2—C1—H1B	109.1	C20—C21—H21A	109.5
H1A—C1—H1B	107.8	C20—C21—H21B	109.5
C2—C3—H3A	109.5	H21A—C21—H21B	109.5
C2—C3—H3B	109.5	C20—C21—H21C	109.5
H3A—C3—H3B	109.5	H21A—C21—H21C	109.5
C2—C3—H3C	109.5	H21B—C21—H21C	109.5
H3A—C3—H3C	109.5	C20—C22—H22A	109.5
H3B—C3—H3C	109.5	C20—C22—H22B	109.5
C3—C2—C4	111.80 (19)	H22A—C22—H22B	109.5
C3—C2—C1	112.21 (18)	C20—C22—H22C	109.5
C4—C2—C1	108.81 (17)	H22A—C22—H22C	109.5
C3—C2—H2A	108.0	H22B—C22—H22C	109.5
C4—C2—H2A	108.0	C24—C23—N4	116.0 (2)
C1—C2—H2A	108.0	C24—C23—C24'	27.3 (2)
C2—C4—H4A	109.5	N4—C23—C24'	111.1 (2)
C2—C4—H4B	109.5	C24—C23—H23A	108.3
H4A—C4—H4B	109.5	N4—C23—H23A	108.3
C2—C4—H4C	109.5	C24'—C23—H23A	131.6
H4A—C4—H4C	109.5	C24—C23—H23B	108.3
H4B—C4—H4C	109.5	N4—C23—H23B	108.3
N1—C5—C6	113.66 (17)	C24'—C23—H23B	85.8
N1—C5—H5A	108.8	H23A—C23—H23B	107.4
C6—C5—H5A	108.8	C24—C23—H23C	126.2
N1—C5—H5B	108.8	N4—C23—H23C	109.4
C6—C5—H5B	108.8	C24'—C23—H23C	109.4
H5A—C5—H5B	107.7	H23A—C23—H23C	82.0
C7—C6—C8	111.48 (19)	H23B—C23—H23C	26.8
C7—C6—C5	112.55 (18)	C24—C23—H23D	82.9
C8—C6—C5	107.27 (18)	N4—C23—H23D	109.4
C7—C6—H6	108.5	C24'—C23—H23D	109.4
C8—C6—H6	108.5	H23A—C23—H23D	28.7
C5—C6—H6	108.5	H23B—C23—H23D	130.0
C6—C7—H7A	109.5	H23C—C23—H23D	108.0
C6—C7—H7B	109.5	C23—C24—C26	109.5 (3)
H7A—C7—H7B	109.5	C23—C24—C25	108.2 (6)
C6—C7—H7C	109.5	C26—C24—C25	106.9 (7)
H7A—C7—H7C	109.5	C23—C24—H24	110.7
H7B—C7—H7C	109.5	C26—C24—H24	110.7
C6—C8—H8A	109.5	C25—C24—H24	110.7
C6—C8—H8B	109.5	C24—C25—H25A	109.5
H8A—C8—H8B	109.5	C24—C25—H25B	109.5
C6—C8—H8C	109.5	H25A—C25—H25B	109.5
H8A—C8—H8C	109.5	C24—C25—H25C	109.5
H8B—C8—H8C	109.5	H25A—C25—H25C	109.5
N1—C9—N2	115.45 (17)	H25B—C25—H25C	109.5
N1—C9—S1	125.80 (15)	C24—C26—H26A	109.5
N2—C9—S1	118.74 (15)	C24—C26—H26B	109.5
O1—C10—N2	122.87 (18)	H26A—C26—H26B	109.5
O1—C10—C11	121.82 (18)	C24—C26—H26C	109.5
N2—C10—C11	115.26 (16)	H26A—C26—H26C	109.5
C16—C11—C12	119.89 (18)	H26B—C26—H26C	109.5
C16—C11—C10	121.58 (17)	C26'—C24'—C25'	107.7 (7)
C12—C11—C10	118.41 (17)	C26'—C24'—C23	114.8 (4)
C13—C12—C11	120.28 (18)	C25'—C24'—C23	103.6 (5)
C13—C12—H12	119.9	C26'—C24'—H24'	110.1
C11—C12—H12	119.9	C25'—C24'—H24'	110.1
C12—C13—C14	119.94 (18)	C23—C24'—H24'	110.1
C12—C13—H13	120.0	C24'—C25'—H25D	109.5
C14—C13—H13	120.0	C24'—C25'—H25E	109.5
C13—C14—C15	120.12 (18)	H25D—C25'—H25E	109.5
C13—C14—H14	119.9	C24'—C25'—H25F	109.5
C15—C14—H14	119.9	H25D—C25'—H25F	109.5
C16—C15—C14	119.86 (17)	H25E—C25'—H25F	109.5
C16—C15—C17	122.16 (17)	C24'—C26'—H26D	109.5
C14—C15—C17	117.94 (17)	C24'—C26'—H26E	109.5
C15—C16—C11	119.89 (17)	H26D—C26'—H26E	109.5
C15—C16—H16	120.1	C24'—C26'—H26F	109.5
C11—C16—H16	120.1	H26D—C26'—H26F	109.5
O2—C17—N3	123.74 (17)	H26E—C26'—H26F	109.5
O2—C17—C15	121.87 (17)		
			
C9—N1—C1—C2	80.0 (2)	C10—C11—C16—C15	175.89 (17)
C5—N1—C1—C2	−103.8 (2)	C18—N3—C17—O2	12.4 (3)
N1—C1—C2—C3	62.2 (2)	C18—N3—C17—C15	−167.52 (17)
N1—C1—C2—C4	−173.59 (18)	C16—C15—C17—O2	142.2 (2)
C9—N1—C5—C6	96.8 (2)	C14—C15—C17—O2	−35.2 (3)
C1—N1—C5—C6	−79.3 (2)	C16—C15—C17—N3	−37.8 (3)
N1—C5—C6—C7	−57.2 (2)	C14—C15—C17—N3	144.76 (18)
N1—C5—C6—C8	179.81 (17)	C19—N4—C18—N3	172.06 (16)
C1—N1—C9—N2	−173.21 (16)	C23—N4—C18—N3	−13.9 (3)
C5—N1—C9—N2	10.8 (3)	C19—N4—C18—S2	−9.2 (3)
C1—N1—C9—S1	8.1 (3)	C23—N4—C18—S2	164.81 (15)
C5—N1—C9—S1	−167.84 (15)	C17—N3—C18—N4	−68.6 (2)
C10—N2—C9—N1	68.0 (2)	C17—N3—C18—S2	112.56 (18)
C10—N2—C9—S1	−113.25 (18)	C18—N4—C19—C20	−108.5 (2)
C9—N2—C10—O1	−1.9 (3)	C23—N4—C19—C20	77.1 (2)
C9—N2—C10—C11	−179.25 (17)	N4—C19—C20—C22	60.0 (2)
O1—C10—C11—C16	−148.7 (2)	N4—C19—C20—C21	−177.34 (17)
N2—C10—C11—C16	28.7 (3)	C18—N4—C23—C24	−65.4 (3)
O1—C10—C11—C12	27.3 (3)	C19—N4—C23—C24	108.9 (3)
N2—C10—C11—C12	−155.33 (18)	C18—N4—C23—C24'	−94.8 (3)
C16—C11—C12—C13	−1.2 (3)	C19—N4—C23—C24'	79.6 (3)
C10—C11—C12—C13	−177.27 (18)	N4—C23—C24—C26	−56.3 (4)
C11—C12—C13—C14	1.7 (3)	C24'—C23—C24—C26	29.6 (5)
C12—C13—C14—C15	−0.9 (3)	N4—C23—C24—C25	−172.5 (8)
C13—C14—C15—C16	−0.3 (3)	C24'—C23—C24—C25	−86.6 (10)
C13—C14—C15—C17	177.14 (18)	C24—C23—C24'—C26'	−58.2 (6)
C14—C15—C16—C11	0.8 (3)	N4—C23—C24'—C26'	47.9 (4)
C17—C15—C16—C11	−176.57 (17)	C24—C23—C24'—C25'	59.0 (10)
C12—C11—C16—C15	0.0 (3)	N4—C23—C24'—C25'	165.1 (8)

### Hydrogen-bond geometry (Å, º)

**Table:**

*D*—H···*A*	*D*—H	H···*A*	*D*···*A*	*D*—H···*A*
N2—H2···S2^i^	0.88	2.62	3.4745 (17)	163
N3—H3···S2^i^	0.88	2.54	3.3870 (18)	162

Symmetry code: (i) −*x*+1, −*y*+1, −*z*+1.
